# An Approach towards a GMP Compliant In-Vitro Expansion of Human Adipose Stem Cells for Autologous Therapies

**DOI:** 10.3390/bioengineering7030077

**Published:** 2020-07-20

**Authors:** Valentin Jossen, Francesco Muoio, Stefano Panella, Yves Harder, Tiziano Tallone, Regine Eibl

**Affiliations:** 1 Institute of Chemistry and Biotechnology, Zurich University of Applied Sciences, 8820 Wädenswil, Switzerland; regine.eibl@zhaw.ch; 2 Foundation for Cardiological Research and Education (FCRE), Cardiocentro Ticino Foundation, 6807 Taverne, Switzerland; francesco.muoio@cardiocentro.org (F.M.); stefano.panella@cardiocentro.org (S.P.); tiziano.tallone@cardiocentro.org (T.T.); 3 Department of Plastic, Reconstructive and Aesthetic Surgery, Ente Ospedaliero Cantonale (EOC), 6900 Lugano, Switzerland; yves.harder@eoc.ch; 4 Faculty of Biomedical Sciences, Università della Svizzera Italiana, 6900 Lugano, Switzerland

**Keywords:** human adipose stem cells (hASCs), serum- and xeno-free conditions, UrSuppe stem cell culture medium, autologous therapy, kinetic growth modeling, segregated and unstructured growth model

## Abstract

Human Adipose Tissue Stem Cells (hASCs) are a valuable source of cells for clinical applications (e.g., treatment of acute myocardial infarction and inflammatory diseases), especially in the field of regenerative medicine. However, for autologous (patient-specific) and allogeneic (off-the-shelf) hASC-based therapies, in-vitro expansion is necessary prior to the clinical application in order to achieve the required cell numbers. Safe, reproducible and economic in-vitro expansion of hASCs for autologous therapies is more problematic because the cell material changes for each treatment. Moreover, cell material is normally isolated from non-healthy or older patients, which further complicates successful in-vitro expansion. Hence, the goal of this study was to perform cell expansion studies with hASCs isolated from two different patients/donors (i.e., different ages and health statuses) under xeno- and serum-free conditions in static, planar (2D) and dynamically mixed (3D) cultivation systems. Our primary aim was I) to compare donor variability under in-vitro conditions and II) to develop and establish an unstructured, segregated growth model as a proof-of-concept study. Maximum cell densities of between 0.49 and 0.65 × 10^5^ hASCs/cm^2^ were achieved for both donors in 2D and 3D cultivation systems. Cell growth under static and dynamically mixed conditions was comparable, which demonstrated that hydrodynamic stresses (*P/V =* 0.63 W/m^3^, *τ_nt_* = 4.96 × 10^−3^ Pa) acting at *N_s1u_* (49 rpm for 10 g/L) did not negatively affect cell growth, even under serum-free conditions. However, donor-dependent differences in the cell size were found, which resulted in significantly different maximum cell densities for each of the two donors. In both cases, stemness was well maintained under static 2D and dynamic 3D conditions, as long as the cells were not hyperconfluent. The optimal point for cell harvesting was identified as between cell densities of 0.41 and 0.56 × 10^5^ hASCs/cm^2^ (end of exponential growth phase). The growth model delivered reliable predictions for cell growth, substrate consumption and metabolite production in both types of cultivation systems. Therefore, the model can be used as a basis for future investigations in order to develop a robust MC-based hASC production process for autologous therapies.

## 1. Introduction

The successful development and application of cell-based therapies has the potential to treat a number of currently incurable diseases and to improve patient care. It is therefore not surprising that many research activities [1,2] are taking place all over the world in the field of regenerative medicine. However, despite the progress in this field, there are a number of challenges that remain before cell-based therapies can be performed more routinely in clinical practice.

Human Adipose Tissue Stem Cells (hASCs) have demonstrated their potential to target a number of currently incurable clinical conditions [2]. This is not surprising since adipose tissue has recently been discovered to be a novel abundant source of adult stem cells, which can be collected by minimally invasive, low risk procedures for the donors/patients and processed by different techniques [3,4,5]. Moreover, results from recently performed clinical trials have indicated possible applications in the treatment of acute myocardial infarction, stroke and a host of inflammatory and immune disorders [6]. Human ASCs are also gaining increasing interest in plastic and reconstructive surgical procedures, where a trend towards stem cell-based tissue-engineering strategies is evident. However, the majority of these clinical applications require in-vitro expansion of the cells to deliver an effective therapeutic dose. The intention of the in-vitro expansion step is to manufacture a sufficient number of hASCs under Good Manufacturing Practice (GMP) conditions and in a cost-effective manner [7,8,9,10]. The processing of hASCs must be performed in accordance with the Directive 2003/94/EC for cell-based medicinal products [11]. In general, hASC-based therapies can be broadly divided into two categories: patient-specific therapies (autologous) and off-the-shelf therapies (allogeneic). From an economic point of view, the allogeneic therapy approach seems to be the most attractive option at present [12,13,14]. However, a crucial factor for the economic success of allogeneic cell-based therapies in terms of affordability will depend on whether the patient receiving the stem cell therapy will require immunosuppressive medication. A combined treatment with immunosuppressive drugs will significantly increase the overall life cycle cost of the treatment. In contrast, autologous therapies require careful consideration of regulatory challenges as well as the distribution and delivery of a safe and effective cell-based therapeutic. Furthermore, it is crucial to consider how a cell therapy manufacturing process can be developed to consistently manufacture products from multiple patients/donors [15]. Therefore, technical and biological characterizations of different cultivation technologies, different donors and other biological aspects are important and will support the development of descriptive and predictive models in the future. Achieving consistency and reproducibility in the manufacture of medicinal products is a key requirement for regulatory approval [16,17] and can be achieved to some extent by a reduction in process variations. A key aspect in reducing process variation is the elimination of fetal bovine serum (FBS) in the cell culture medium [18]. Various studies have already shown that serum-free cell culture media can be used in combination with stirred bioreactors and Microcarrier (MC) technology [19,20,21,22] in order to expand human mesenchymal stem cells (hMSCs). In addition to the MC-based expansion technology, hollow fiber bioreactors are also frequently used for the hMSC expansion, with which total cell densities of up to 10^9^ hMSC can be achieved [23,24]. In contrast to the MC-based expansion, hMSC cell growth occurs inside the hollow fibers, which are permanently flown through with cell culture medium. However, cell harvest could be problematic in these systems and must be carefully developed based on the expansion process. Amini et al. [25] developed a static, wicking matrix bioreactor that provides a thing film of medium that drips onto cells on the scaffold. They used this new bioreactor concept successfully for the expansion of hiPSC-derived pancreatic cells for the production of insulin. Thus, such new bioreactor concepts are also interesting for the expansion of hMSCs.

In contrast to traditional planar and static cultivation systems, MCs (typically in the range of 100–300 µm) provide a surface on which the strictly adherent hASCs can grow in stirred and instrumented bioreactors. The MCs consist of different materials (e.g., polystyrene and gelatin), including synthetic/organic or natural polymers that are synthesized with different porosities and topographies. The careful selection and tuning of the MCs and the serum-free cell culture medium is important and has an influence on the success of in-vitro cultivation.

The aim of this proof-of-concept study was to perform cell expansion experiments with hASCs isolated from two different patients/donors (i.e., different age and health status) under xeno- and serum-free conditions in static, planar (2D) and dynamically mixed (3) cultivation systems. In so doing, we (I) compared the donor variability under in-vitro conditions in two different cultivation systems and (II) developed and established an unstructured, segregated growth model for future investigations. However, due to the limited accessibility of the donor/patient material, only two donors were considered in the present study in order to establish first versions of the growth model. Special emphasis was placed on determining growth-related parameters (i.e., parameters for growth rate and metabolic flux) and comparing cell-specific Critical Quality Attributes (CQAs) during the processing of the hASCs under the static 2D and dynamic 3D process conditions. The growth-related parameters were subsequently used to establish a mathematical growth model for donor-dependent cell growth description, substrate consumption and metabolite production under static 2D and dynamic 3D process conditions.

## 2. Materials and Methods

### 2.1. Procurement of Subcutaneous Adipose Tissue from Human Donors 

The human adipose tissue samples used in this study (*n* = 2 donors, referred to as 080 and 085) were obtained from tissue excess originating from surgical interventions performed at the Department of Plastic, Reconstructive and Aesthetic Surgery at the Ospedale Regionale di Lugano (Switzerland). All patients who donated their adipose tissue provided written agreement in compliance with the directives of the local Ethics Committee of the Canton of Ticino (Switzerland), which approved the project and its procedures (project reference number: CE 2915).

The cellular sources used in this study originate from subcutaneous adipose tissue harvested from the abdominal region of female patients undergoing autologous breast reconstruction under general anesthesia. Firstly, depending on the position of the deep inferior epigastric artery and its perforating vessels (DIEP-flap), a symmetrical diamond-shaped abdominal flap was dissected between the umbilicus and the pubis. Any excess subcutaneous adipose tissue, not used for breast reconstruction, was packed into two sterile bags to avoid any contamination and was delivered for further processing of the tissue. The adipose tissue samples were stored at room temperature and processed within 24 h [26] to obtain the Stromal Vascular Fraction (SVF).

### 2.2. Isolation and Establishment of a Serum-Free hASC Culture

The extraction of the SVF from human adipose tissue and the in-vitro expansion and cryopreservation of the isolated hASCs was performed in accordance with the ethical principles outlined in the Declaration of Helsinki and in compliance with the directives of the Ethics Committee of the Canton of Ticino (Switzerland). The isolated tissue samples were firstly separated from the skin tissue, washed in PBS and homogenized in a blender for 10–15 s (100–400 g of fat tissue). After this initial step, the tissue was digested for 45 min at 37 °C with 0.28 Wünsch Unit/mL of Collagenase AB [27] (Worthington Biochemical Corp., Lakewood, NJ, USA). The enzymatic reaction was stopped by the addition of PBS supplemented with 1% human albumin (CSL Behring AG, Bern, Switzerland). After separating the aqueous phase from the lipid phase, the aqueous phase was collected in a new sterile tube. The cells were subsequently centrifuged and filtered to obtain a fresh SVF.

In order to characterize the SVF, the cells were stained with anti-CD34-BV650, anti-CD45-PC7, anti-CD73-FITC (BioLegend, San Diego, CA, USA), anti-CD146-PE, anti-CD36-APC (Miltenyi BioTech, Bergisch Gladbach, Germany), 7-amino-actinomycin D (7-AAD) (Becton Dickinson, Franklin Lakes, NJ, USA) and Syto40 (Life Technologies from Thermo Fisher Scientific, Waltham, MA, USA). All of the antibodies were titrated to optimize the signal–to–noise ratio and used at a specific concentration (further information can be found in “Supplementary Materials Table S2”). After 20 min of incubation, the erythrocytes were lysed with 1 mL of VersaLyse solution (Beckman Coulter Inc., Brea, CA, USA). A Forward Scatter Time-of-Flight channel was used to select single cell events, Syto40 DNA marker was used to exclude cellular debris and 7-AAD was used to discriminate between dead and living cells. Cells were acquired using a Cytoflex flow cytometer (Beckman Coulter Inc., Brea, CA, USA). The ASC cell population was defined as CD45^−^, CD146^−^, CD36^−^, CD34^+^ and CD73^+^.

After characterization, cells were seeded at a density of 30,000 ASCs/cm^2^ in fibronectin precoated plates (Corning Inc., New York City, NY, USA) with our chemically defined serum- and xeno-free stem cell culture medium, called UrSuppe. The stem cell culture medium was changed every 2–3 days, always keeping 50% of the conditioned medium, until the cells reached a confluency of 80–90%. For passaging, the cells were detached from the growth surface by incubating them for 2 min at 37 °C in TrypLE Select [28] (Life Technologies from Thermo Fisher Scientific, MA, USA). After discarding the supernatant, the cells were resuspended in UrSuppe and passaged or used for other experimental investigations in this study.

### 2.3. hASC Growth Characterization under Planar, Static Conditions (2D Monolayer Expansion)

2D growth characterization of previously isolated hASCs was performed in precoated T_25_-flasks (5 µg/cm^2^ r-fibronectin; Sigma Aldrich, St. Louis, MO, USA) with the UrSuppe stem cell culture medium (5 mL). For this purpose, the cryopreserved, patient-derived hASCs (P1, 080-PDL_cum._ 3.9, 085-PDL_cum._ 3.7) were thawed and precultured in T_75_-flasks (10,000 hASCs/cm^2^; 37 °C, 5% CO_2_, 80% rH) in order to achieve the required cell numbers to inoculate 22 × T_25_-flasks per donor (P2, 080-PDL_cum._ 6.3, 085-PDL_cum._ 6.5, 10,000 cells/cm^2^). The hASC growth characteristics were assessed over 11 days by harvesting two T_25_-flasks per donor (2 mL TrypLE Select at 37 °C, 2 min) every day. The cell density, substrate and metabolite measurements were carried out using a NucleoCounter NC-200 (Chemometec, Allerod, Denmark) and a Cedex Bio (Roche Diagnostics, Rotkreuz, Switzerland), respectively. In addition to standard T_25_-flasks, T_25_-flasks equipped with pH and DO sensor spots (PreSens, Regensburg, Germany) were also inoculated in parallel for each donor in order to assess the pH and DO profiles during cell growth (37 °C, 5% CO_2_, 80% rH). In each case, partial medium exchanges of 40% and 60% were performed for each donor on days 4 and 8.

### 2.4. hASC Growth Characterization under Dynamically Mixed Conditions (Microcarrier-Based Expansion)

3D growth characterization was performed for each donor (P3, 080-PDL_cum._ 11.5, 085-PDL_cum._ 11.6) using fibronectin-coated polystyrene beads (ProNectin^®^ F-COATED, Pall SoloHill, New York City, NY, USA) in 125 mL disposable Corning spinner flasks (=100 mL UrSuppe). An initial cell density of 15,000 cells/cm^2^ (=54,000 cells/mL) and a Microcarrier (MC) concentration of 10 g/L (=1 g, 360 cm^2^) were used to inoculate the spinner flasks. The MC concentration of 10 g/L was defined based on previous investigations by Schirmaier et al. [29] and Jossen et al. [1,30]. The cell inoculum was prepared in T_75_-flasks coated with r-fibronectin (5 µg/cm^2^) and with cells from P1 (=080-PDL_cum._ 3.9, 085-PDL_cum._ 3.7). Before inoculation, the MCs were prepared and sterilized according to the vendor recommendations one day before usage. After cell inoculation, a static cell attachment phase of 24 h was performed in a cell culture incubator (37 °C, 5% CO_2_, 80% rH) to allow the cells to attach to the MC surface. After the static attachment phase, the culture was continuously stirred at 49 rpm. The selected impeller speed, which corresponded to the *N_s1u_* criterion for 10 g/L MCs in the 125 mL disposable Corning spinner flask, was defined based on experimental and numerical fluid flow investigations by Kaiser et al. [31] and Jossen et al. [1,30]. The *N_s1u_* suspension criterion defines the lower limit of *N_s1_* (=*N_js_*), meaning that some MC beads are still in contact with the reactor bottom, but none of them were at rest [32]. On day 5, a partial medium exchange of 50% was performed. For this purpose, the impeller was switched off and the MCs were allowed to settle. Fifty percent of the working volume was replaced with fresh preheated UrSuppe stem cell culture medium, and the impeller was restarted. No MC feeds were performed during the cultivations.

Off-line samples were taken daily to measure substrate and metabolite concentrations (Glc, Lac and Amn) with a Cedex Bio (Roche Diagnostics, Rotkreuz, Switzerland). After the cells had been detached from the MC surface by the enzymatic treatment (15 min with TrypLE Select), the hASC cell number was measured using a NucleoCounter NC-200. The measured cell specific values were used to calculate the growth-related parameters as described in Section 2.6. In addition to the cell measurements, 1 mL of the MC-cell suspension was fixed immediately after sampling with a 3% paraformaldehyde solution for 4′,6-diamidin-2-phenyliondol (DAPI) staining.

### 2.5. Cell Analytics

#### 2.5.1. Flow Cytometric Analysis

Flow cytometric measurements were performed at the end of the growth characterization experiments (10th day of cultivation): 2D monolayer and MC-based expansion. The flow cytometric measurements contained different mixtures of the following antibodies: CD26-FITC, CD73-FITC, CD90-APC, CD105-PE (BioLegend, San Diego, CA, USA), CD36-APC, CD146-PE (Miltenyi Biotec, Bergisch Gladbach, Germany), CD55-BV421 (Becton Dickinson, Franklin Lakes, NJ, USA) and CD54-PE (Thermo Fisher Scientific, Waltham, USA). All of the antibodies were titered in advance in order to improve the signal-to-noise ratio; the final measurements were carried out with 50 ng/test (respective mAbs and Isotype controls). A Zombie Yellow™ Fixable Viability Kit (BioLegend, San Diego, CA, USA) was used to distinguish between live and dead cells after fixation (1% paraformaldehyde in DPBS for 1 min at RT). For the staining procedure, 50,000 cells in 100 µL FACS buffer (PBS supplemented with 1% albumin and 50 ng/µL human immunoglobulin, Privigen Immunogobulin, CSL Behring AG, Bern, Switzerland) were pipetted into a well, gently mixed and subsequently incubated in the dark for 15 min at room temperature. After the incubation step, the samples were diluted with 100 µL FACS buffer. Sample acquisition and analysis were performed using a Cytoflex flow cytometer (Beckam Coulter Inc., Brea, CA, USA) and Kaluza analysis software. The spectral spill-over from the different fluorochromes was assessed by spectral compensation of the individual fluorescence channels. For this purpose, single stained control particles (VersaComp Antibody Capture Bead Kit, Beckman Coulter, Brea, CA, USA) or cells in combination with the different fluorochromes were used. The compensation matrix was automatically calculated using the dedicated software function integrated into the Kaluza analysis software. Flow cytometer functionality, including the control of the optical alignment and fluidics, was verified routinely with fluorospheres (CytoFLEX Daily QC fluorospheres, Beckman Coulter, Brea, CA, USA). Further information about the different antibodies can be found in “Supplementary Materials Table S2”.

#### 2.5.2. RT-qPCR Analysis

RT-qPCR measurements were carried out at different times (day 1, day 5 and day 10) after daily harvesting of the hASCs. The different RT-qPCR measurement times represent distinct phases of cell proliferation; day 1: start of cell proliferation, day 5: exponential cell growth and day 10: plateau due to cellular confluence (end of cultivation). For this purpose, RNAs were extracted from the cell pellets or from the MCs covered with cells by using a Nucleospin^®^RNA kit (Macherey-Nagel, Düren, Germany). The RNA purification process included an on-column digestion step with DNase I and was performed according to the manufacturer instructions. The RNA purity and quantity was assessed with a NanoDrop microvolume spectrophotometer (Thermo Fisher, Waltham, MA, USA) and the total RNA integrity was periodically verified by agarose gel analysis. cDNA was obtained from 900 ng RNA using a GoScript^TM^ Reverse Transcription System (Promega, CA, USA). Detailed information about the protocol can be found in “Supplementary Materials Table S3”. RT-qPCR of the *PREF1, SOX9, WISP1, WISP2, NOTCH1, DLL1, CD26, CD55, CD248, CD142, ZP521, ZFP423, PPARG, DKK1, RUNX2, CD34, CD36* and *CD146* genes was performed using 20 ng cDNA for each gene of interest and a SsoAdvanced^TM^ Universal SYBR^®^ Green Supermix kit (Biorad, Hercules, CA, USA) in combination with a CFX Connect System for signal detection. An overview of the different primer sequences is shown in Table 1, where ACTB was used as an internal control for all measurements. Each primer pair product was checked for proper amplification using agarose gel electrophoresis and only single sharp bands of the expected size were used for further analysis. The RT-qPCR process was divided into 5 phases: (I) initial denaturation (95 °C, 120 s), (II) cycle denaturation (95 °C, 5 s), (III) cycle annealing and extension (60 °C, 20 s), (IV) final denaturation (95 °C, 5 s) and (V) melting curve (65–95 °C, 18 min), where phases II and III were repeated 40 times in the sequence. The resulting data were analyzed using CFX software in order to evaluate the ΔΔCt values, which were normalized using the value of the housekeeping gene ACTB as the reference gene. The relative fold changes of the analyzed genes are related to the beginning of the culture (day 1). 

### 2.6. Determination of Cell Biological Kinetic Parameters: Growth Dynamics and Metabolic Activity

Based on regular measurements of cell density and substrate/metabolite concentration, growth-dependent parameters were calculated for the planar and MC-based cultivations as follows:

(I) Specific growth rate (μ):

(1) $$\mu =\frac{ln\left(X_{A}\left(t\right)\right)-ln\left(X_{A}\left(0\right)\right)}{\Delta t}$$

where μ is the net specific growth rate (d^−1^), *X_A_(t)* and *X_A_(0)* are the cell numbers (cells/cm^2^) at the end and the beginning of the exponential growth phase, respectively, and *t* is the time (d).

(II) Doubling time (*t_d_*):

(2) $$t_{d}=\frac{ln\left(2\right)}{\mu }$$

where *t_d_* is the doubling time, *ln(2)* the binary logarithm of 2 and *µ* the specific cell growth rate.

(III) Population Doubling Level (*PDL*):

(3) $$PDL=\frac{1}{log\left(2\right)}\cdot log\left(\frac{X_{A}\left(t\right)}{X_{A}\left(0\right)}\right)$$

where *PDL* is the number of population doublings, and *X_A_(0)* and *X_A_(t)* are the cell numbers (cells/cm^2^) at the beginning and the end of the cultivation, respectively.

(IV) Expansion factor (*EF*):

(4) $$EF=\frac{X_{A}\left(t_{max}\right)}{X_{A}\left(t=1\right)}$$

where *EF* is the expansion factor and *X_A_(_tmax_)* is the maximum cell number and *X_A_(_t=1_)* is the cell number on day 1 (i.e., after cell attachment phase).

(V) Lactate yield from glucose (*Y_Lac/Glc_*):

(5) $$Y_{Lac/Glc}=\frac{\Delta Lac}{\Delta Glc}$$

where *Y_Lac/Glc_* is the lactate yield from glucose, Δ*Lac* is the lactate production over a specific time period and Δ*Glc* is the glucose consumption over the same time period (=exponential growth phase).

(VI) Specific metabolic flux (*q_met_*):

(6) $$q_{met}=\left(\frac{\mu }{X_{A}\left(t\right)}\right)\left(\frac{C_{met}\left(t\right)-C_{met}\left(0\right)}{e^{\mu t}-1}\right)$$

where *q_met_* is the net specific metabolite consumption or production rate (for Glc, Lac and Amn), *µ* is the specific cell growth rate (d^−1^), *X_A_(t)* is the cell number (cells/cm^2^) at the end of the exponential growth phase, *C_met_(t)* and *C_met_(0)* are the metabolite concentrations (mmol/L) at the end and the beginning of the exponential growth phase, respectively, and *t* is the time (d).

### 2.7. Modelling of hASC Growth Kinetics in 2D Culture Systems (T_25_-Flasks) 

Based on the findings from the static, planar growth experiments, an unstructured, segregated, simplistic growth model was developed and used to describe the hASC growth kinetics in the T_25_-flask cultures. A comparable model approach has already been successfully used by Jossen et al. [1] to simulate the anchorage-dependent growth of hASCs on MCs during serum-reduced (5% FBS) expansion in single-use spinner flasks. The same model approach as that employed by Jossen et al. [1] was also used with only minor modifications to simulate MC-based hASCs growth kinetics in this study. Detailed information about the MC-based growth model can be found in Jossen et al. [1] and in “Supplementary Materials”. 

**Figure 1:**
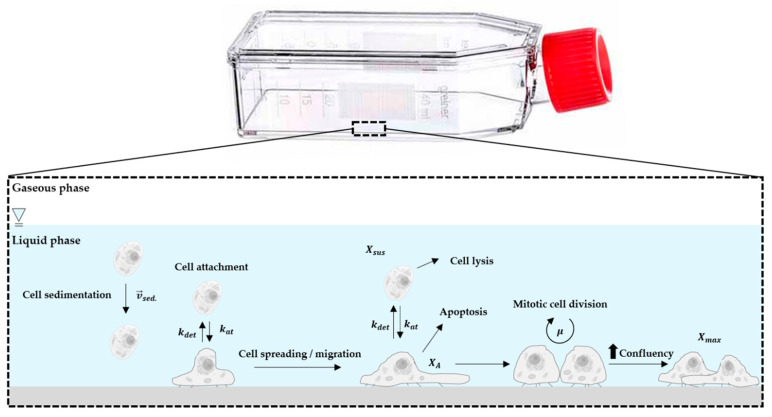
Principle of the growth model and influencing factors.

The general concept for the growth model and the factors that influence the T_25_-flask cultures are shown in Figure 1. Since hASC growth is anchorage-dependent, possible formation of spheroids in the suspension was not considered in the model. This simplification was justified since no spheroid formation was observed in any of the 2D cultivations that employed an appropriate surface coating (data not shown). Thus, it can be assumed that cells in suspension do not contribute to an increase in the overall cell number, with cell growth restricted to the planar growth surface. To define the starting conditions, it was assumed that initial cell attachment took place during the cell attachment phase, which can be described by the attachment constant *k_at_*. After the cells had attached themselves to the planar growth surface, a short cell adaption phase was considered, before the cells began to proliferate.

The cell adaption phase was considered by introducing the coefficient α(t) (see Equation (7)),

(7) $$\alpha \left(t\right)=\frac{t^{n}}{{t_{l}}^{n}+t^{n}}$$

where $t_{l}$ defines the lag time and the point at which α(t) is half of the maximum. The exponent *n* in Equation (7) affects the slope of f(α(t)). If *n* = 1, α(t) is described by Michaelis–Menten kinetics. Otherwise, a sigmoidal curve is obtained that becomes steeper as *n* increases. Both variables ($t_{l}$ and *n*) were obtained from experimental growth studies using different donor cells.

The specific cell growth rate (*µ*) was calculated based on Monod-type kinetics. Hence, glucose (Glc), lactate (Lac), ammonium (Amn) and the available growth surface (*X_max_*) were considered to be influencing factors (see Equation (8)). However, analysis of microscopic pictures from time lapse microscopic investigations indicated that cell growth restriction based on the maximum available growth surface does not follow a normal Monod-type kinetic (data not shown). This observation can mainly be ascribed to cell migration during cell growth. Thus, the effect of the growth surface restriction term becomes more significant towards the end of the cell growth phase. For this reason, the exponent *n* was also introduced in Equation (8) as a growth surface restriction term.

(8) $$\mu =\mu_{max}\cdot \left(\frac{Glc}{K_{Glc}+Glc}\right)\cdot \left(\frac{K_{Lac}}{K_{Lac}+Lac}\right)\cdot \left(\frac{K_{Amn}}{K_{Amn}+Amn}\right)\cdot \left(\frac{{X_{max}}^{n}-{X_{A}}^{n}}{{X_{max}}^{n}}\right)$$

The cell number on the planar growth surface ($X_{A}$) increased through mitotic cell division and the attachment of cells from the suspension (see Equation (9)). However, this cell number increase was affected by the detachment of hASCs from the planar growth surface, which was accounted for by the detachment constant (*−k_det_*).

(9) $$\frac{dX_{A}}{dt}=\alpha \cdot \mu \cdot X_{A}+k_{at}\cdot \frac{\left({X_{max}}^{n}-{X_{A}}^{n}\right)}{{X_{max}}^{n}}\cdot X_{Sus}-k_{det}\cdot X_{A}$$

Since T_25_-flasks are static systems, *k_det_* is not substantially affected by changing hydrodynamic stresses and can be assumed to be constant. hASC growth in suspension was negligible and therefore changes in cell number were only affected by cell attachment to or detachment from the growth surface (see Equation (10)).

(10) $$\frac{dX_{Sus}}{dt}=k_{det}\cdot X_{A}-k_{at}\cdot \frac{\left({X_{max}}^{n}-{X_{A}}^{n}\right)}{{X_{max}}^{n}}\cdot X_{Sus}$$

Glucose consumption was assumed to be limited by the glucose concentration itself (see Equation (11)). In other words, glucose consumption was the result of glucose uptake by the mitotic cells and the maintenance metabolism of the mitotic and non-mitotic cells ($X_{V}$). A step response ($\delta_{Glc}$) was implemented in Equation (11) to avoid negative glucose concentrations, even though it was highly improbable that *Glc* was completely consumed during the culture time. This was mainly due to the frequent partial medium exchanges and the theoretically low maximum ratio of cells to *Glc* in the T_25_-flasks.

(11) $$\frac{dGlc}{dt}=-\frac{1}{Y_{\frac{X}{Glc}}}\cdot \alpha \cdot \mu \cdot \frac{\left({X_{max}}^{n}-{X_{A}}^{n}\right)}{{X_{max}}^{n}}\cdot X_{A}-m_{Glc}\cdot \delta_{Glc}\cdot X_{V}$$

L-glutamine (Gln) consumption was not considered in this model, since metabolic measurements indicated that Gln was not a limiting factor in the T_25_-flask cultures. Moreover, UltraGlutamine (L-alanyl-L-glutamine) was used in the UrSuppe stem cell culture medium, which had undergone a series of complex degradation steps (i.e., (I): cleavage by extracellular peptidases, (II) degradation of free L-glutamine or absorption into the cells and metabolization). The production of lactate (Lac) and ammonium (Amn) was accounted for by Equations (12) and (13).

(12) $$\frac{dLac}{dt}=q_{Lac}\cdot X_{A}\cdot \alpha +p_{Lac}\cdot X_{V}$$

(13) $$\frac{dAmn}{dt}=q_{Amn}\cdot X_{A}\cdot \alpha +p_{Amn}\cdot X_{V}$$

All growth-related simulations were performed using MATLAB 2019a (MathWorks Inc., Natick, MA, USA). The set of model equations were solved using the ode15s solver in MATLAB. 

## 3. Results and Discussion

### 3.1. Isolation of hASCs from Subcutaneous Adipose Tissue (SAT)

The SVF obtained from human subcutaneous adipose tissue is a heterogeneous mixture of cells, which are isolated by enzymatic dissociation. In general, adipocytes represent roughly two-thirds of the total cells extracted and the rest are blood-derived cells, vascular cells, endothelial cells, smooth muscle cells, pericytes, fibroblasts and hASCs. A multiparameter flow cytometric assay was used in the study to determine the absolute cell number for every cell population and to characterize the cells in the SVF. For this purpose, the target hASC population was defined as being positive for CD34^+^ and CD73^+^, and negative for CD36^−^, CD45^−^ and CD146^−^ [33,34]. Table 2 provides an overview of the two patients/donors (080 = healthy patient, 085 = post-chemotherapy patient) investigated in this study and the number of living hASCs isolated from their biopsies. For both investigated cases, the number of isolated hASCs was in the range of 5.7–7.7% of the total living cell population and the fraction of hASCs obtained from donor 085 was 35% greater than from donor 80. Based on the information about the number of live hASCs per patient biopsy, the cells were directly seeded in precoated T-flasks (30,000 cells/cm^2^) with our xeno- and serum-free UrSuppe stem cell culture medium in order to establish P0. 

### 3.2. hASC Growth under Planar, Static Conditions

**Figure 2:**
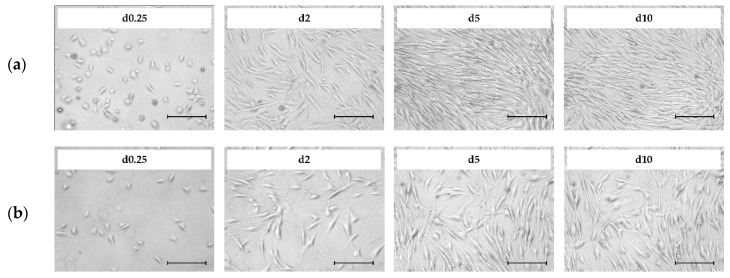
Light microscopic pictures of patient-derived Human Adipose Tissue Stem Cells (hASCs; **a** = donor 080, **b** = donor 085) during cell growth in T_25_-flasks. Scale bar = 275 µm.

Figure 2 shows light microscopic pictures of the patient-derived hASCs (a = donor 080, b = donor 085) during the growth characterization study in the T_25_-flasks. It is clear that cell attachment occurred during the first 4–6 h after cell inoculation. In both cases, ≥98% of the inoculated cells attached to and spread out across the growth surface under the xeno- and serum-free conditions. These cells exhibited typical fibroblast-like or fibroblastoid cell morphology, with minimum and maximum cell diameters in the range of 12–54 µm and 30–291 µm. Interestingly, analysis of the light microscopic pictures showed that the hASCs isolated from donor 085 had a higher average cell area (2480 µm^2^, + 24–30%) compared to those from donor 080 (1810 µm^2^). Therefore, lower maximum cell densities (=cells/cm^2^) can be expected for donor 085, which has an effect on the total cell yield and future process designs. Qualitative analysis of the microscopic pictures showed that the cells began to migrate and proliferate immediately after the cell attachment phase. As expected, cell confluency increased in both cases as a function of the cell number. This resulted in a cell confluency of nearly 80–90% in both cultures after day 5. From day 5 to day 10, the increase in cell confluency slowed down due to the reduced cell proliferation rate, which was caused by the higher frequency of cell contact inhibition. A maximum cell confluency of 95–100% was achieved in both cases by the end of the cultivation studies (=day 10). From a visual point of view, no significant differences in morphology (i.e., shape, granularity) were found between the two donors or during the culture time.

**Figure 3:**
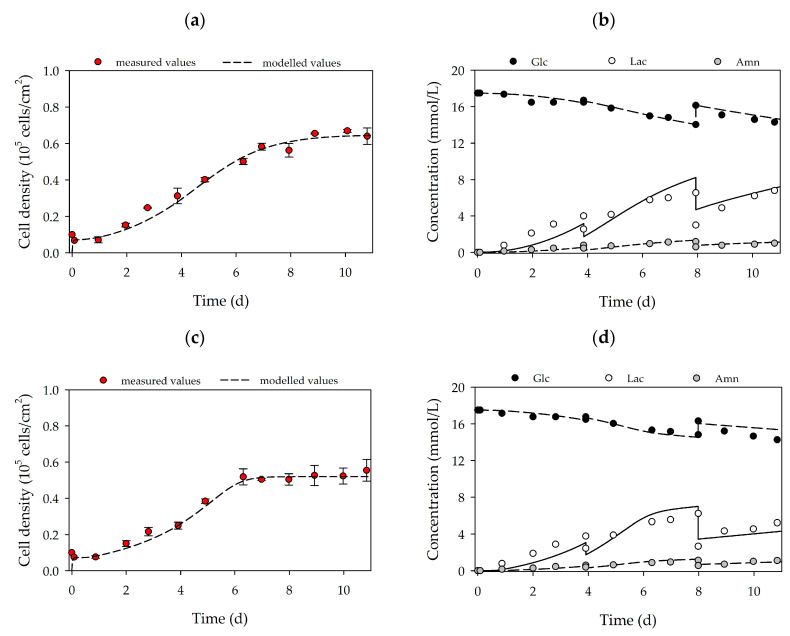
Time-dependent profiles of cell densities (**a**,**c**) and substrate/metabolite concentrations (**b**,**d**) in T_25_-flasks. Donor 080 (upper row) and 085 (lower row). Partial medium exchanges of 40% and 60% were performed on days 4 and 8, respectively. The symbols represent the experimentally measured values collected from offline measurements. The lines represent the simulated time courses.

Figure 3a–d shows a quantitative analysis of cell growth and substrate/metabolite concentrations. It is clear that in both cases, the hASCs followed a classical exponential growth curve, which was characterized by four different growth phases: (I) cell adaption phase, (II) exponential growth phase, (III) cell growth restriction phase and (IV) stationary growth phase. A maximum cell density of 0.65 ± 0.02 × 10^5^ hASCs/cm^2^ (=3.25 ± 0.1 × 10^5^ hASCs/mL) and 0.52 ± 0.02 × 10^5^ hASCs/cm^2^ (=2.60 ± 0.1 × 10^5^ hASCs/mL) was achieved for donors 080 and 085, respectively. Consequently, the peak cell density of the hASCs from donor 080 was 25% higher than for donor 085. As already mentioned, the differences in the maximum cell densities may be explained by the higher average hASC cell areas from donor 085 compared to donor 080. Interestingly, cell size could be a “consequence” of slow cell growth, in which some cells increase their volume by an increased DNA content and/or other macromolecules [35]. However, these observations need to be further investigated in future studies. During the culture period, cell viability was in both cases always > 95%. The maximum cell densities corresponded to maximum PDLs and EFs in the range of 2.79–3.22 and 7.4–9.9, respectively. Hence, the hASC PDL and EF from donor 080 were 15% and 33% higher, respectively, than for the hASCs from donor 085. Due to the metabolic activity of the cells during the growth phase, glucose was consumed, and lactate and ammonium were produced (Figure 3b,d). In both cultures, the glucose concentration did not drop below 14.04 mmol/L due to the regular partial medium exchanges. Maximum lactate and ammonium concentrations were measured in both cultures in the range of 6.2–6.8 mmol/L and 1.13–1.16 mmol/L. Based on data from Higuera et al. [36] and Schop et al. [37,38] lactate and ammonium did not, however, reach growth-inhibiting concentrations (Lac = 25–35 mM, Amn = 2.5 mM). The online measured pH values (data not shown) agreed well with offline measured data (± 1%) and indicated stable pH values in the region of 7.2–7.3 during the entire cultivation. Cellular respiration caused oxygen to be consumed during cell growth. However, the oxygen supply in the T_25_-flasks was not a limiting factor and the DO values did not drop below 80% (data not shown). It is clear that by using the developed growth model (see Figure 3a–d lines), the time courses of the cell densities on the MC surface, and the substrate and metabolite concentrations could be well approximated. As indicated by the light microscopic pictures, only a few cells were observed in the supernatant during the static 2D cultivations. Hence, cell density in the supernatant was negligible (simulation results see “Supplementary Material”). Maximum deviations between the measured and simulated cell densities in both cultures were in the range of 8–16%, while slightly higher deviations of up to 21% were found between the measured and simulated substrate/metabolite concentrations. These higher deviations can be explained by (I) uncertainties in substrate/metabolite measurements, (II) error propagation in the calculation of the specific consumption/production rates and (III) slightly different behavior in the real cellular metabolism. Nevertheless, based on growth-dependent parameters, the model successfully describes and predicts cellular growth, substrate consumption and metabolite production in the static 2D cultivation systems.

Table 3 summarizes the calculated growth-dependent parameters for donors 080 and 085. It is clear that with a specific growth rate of 0.52 d^−1^ (t_d_ = 32 h), the hASCs from donor 080 grew 33% faster than the hASCs from donor 085 (*µ_max_* = 0.39 d^−1^, *t_d_* = 42.7 h). Salzig et al. [20] reported specific growth rates for human bone marrow-derived mesenchymal stem cells (hBM-MSCs) cultivated in a serum-free culture medium in the range of 0.38–0.45 d^−1^ (*t_d_* = 36.9–43.7 h). Comparable specific growth rates (0.31–0.47 d^−1^) were also reported by Heathman et al. [22] for hBM-MSCs from different donors and over different passages. Therefore, specific hASC growth rates obtained in this study were in a comparable range or even slightly higher (+15%). However, a direct comparison of the specific growth rates is critical since the hMSCs were from different tissue sources and donors and were grown in different serum-free cell culture media. Specific glucose consumption rates (*-q_Glc_*) were between 1.35 and 1.98 pmol/cell/d and demonstrated that the glucose was more efficiently metabolized by the cells from donor 080. As a result, hASCs from donor 085 produced more lactate for the same equivalent amount of glucose (*Y_Lac/Glc_*: 1.14 vs. 1.05 mmol/mmol). Ammonium production (0.28–0.32 pmol/cell/d) was comparable in both cultures.

### 3.3. MC-Based hASC Expansion in Single-Use Spinner Flasks

**Figure 4:**
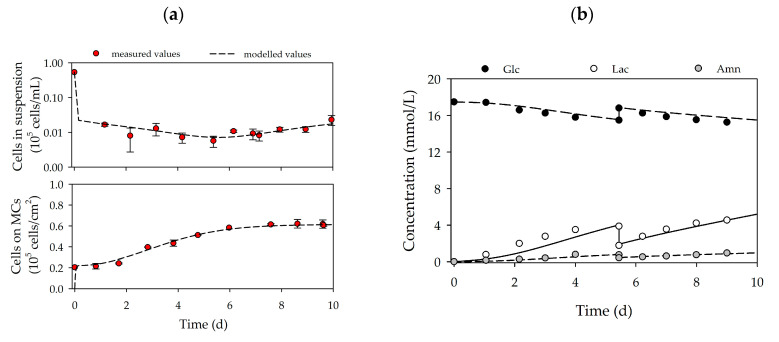

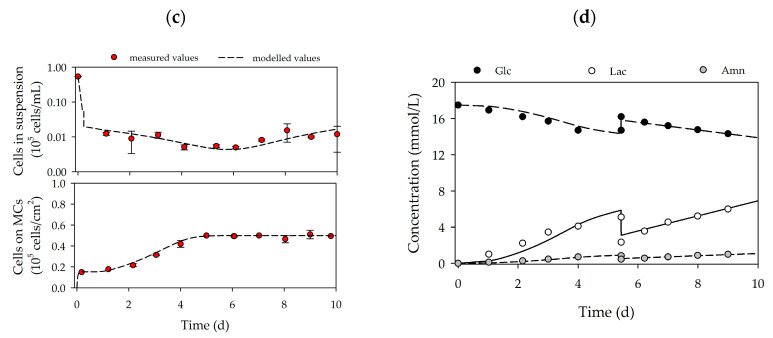
Time-dependent profiles of cell densities (**a**,**c**) and substrate/metabolite concentrations (**b**,**d**) in the Corning spinner flasks. Donor 080 (upper row) and 085 (lower row). A partial medium exchange of 50% was performed on day 5. The symbols represent the experimentally measured values collected by offline measurements. The lines represent the simulated time courses.

Based on the growth-related parameters obtained from the planar growth characterization studies, cell growth was also characterized for dynamic conditions in MC-based cultivations. Figure 4a,d shows the time-dependent profiles of the cell density and the substrate/metabolite concentrations for hASCs from donors 080 (upper row) and 085 (lower row). After the 24 h cell attachment phase, a cell attachment efficiency of 137% (080) and 118% (085) was achieved. These results indicate that a portion of the cell population had already started to divide within the static cell attachment phase. A peak cell density of 0.61 ± 0.01 × 10^5^ hASCs/cm^2^ (=2.16 ± 0.04 × 10^5^ hASCs/mL) was achieved for donor 080. At 0.49 ± 0.01 × 10^5^ hASCs/cm^2^ (=1.76 ± 0.04 × 10^5^ hASCs/mL), the maximum cell density for donor 085 was again lower (−19%), but nonetheless agreed well with the data from the planar 2D cultivations. In both cases, the maximum cell densities in the MC-based cultivations agreed well with those achieved in the planar cultivation systems. In both cultures, maximum PDLs and EFs were in the range of 1.58–1.72 and 3.2–3.3, respectively. During the cultivation, glucose concentrations decreased to 15.2 mmol/L (080) and 14.3 mmol/L (085), meaning glucose was not a limiting factor in either cultivation. As a result of glucose metabolization, lactate concentration increased to maximum values of 4.5 mmol/L (080) and 5.9 mmol/L (085), and ammonium concentrations remained relatively low during the entire cultivation (080-*Amn* = 0.94 mmol/L, 085-Amn = 0.99 mmol/L). Both lactate and ammonium levels were below critical concentrations [36,37,38]. From the time-dependent profiles of Figure 4, it can be seen that the growth model can also be used to describe the growth kinetics and substrate/metabolite profiles in MC-based hASC cultivations. The simulated and measured values differed only slightly. Maximum deviations in cell density of ≤7% were found, and deviations in the substrate/metabolite concentrations were only slightly higher (≤15%). Nonetheless, the model can be used in the future to describe hASC growth kinetics in different cultivation settings.

Table 4 provides an overview of the main growth-dependent parameters calculated for the two different hASC cultivations. The hASCs from the two different donors grew at comparable specific growth rates of between 0.42 and 0.44 d^−1^ (*t_d_* = 37.8–39.6 h). The calculated specific growth rates were in a comparable range to literature data for MC-based expansions of hBM-MSCs in serum-free cell culture media [20,21,22,39]. In both cases, glucose was metabolized less efficiently by the hASCs, although -*q_Glc_* values (1.34–1.96 pmol/cell/d) were comparable to those in the 2D cultures. Therefore, *Y_Lac/Glc_* was in the range of 1.39–1.68 mmol/mmol. The less efficient metabolism of glucose was caused by a higher *q_Lac_*, which might be a consequence of the hydrodynamic stresses acting on the cells in dynamically mixed systems. The rates of ammonium production in both cultures (0.26–0.27 pmol/cell/d) were comparable with those in the 2D culture systems.

**Figure 5:**
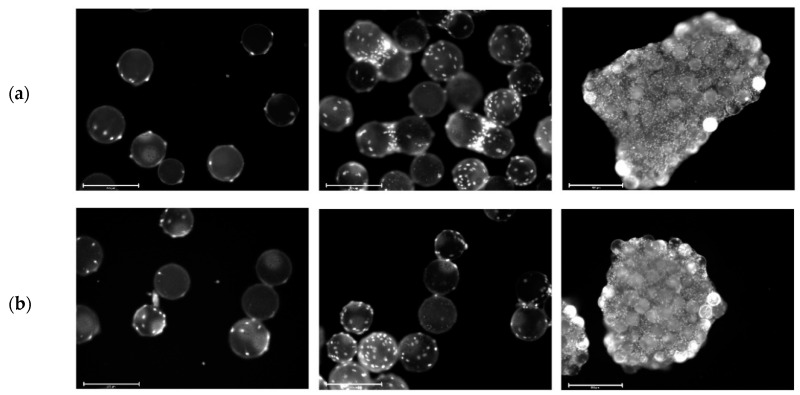
Fluorescence microscopic images during cell growth in Corning spinner flasks. Donors 080 (**a**) and 085 (**b**). DAPI-stained cells on Microcarriers (MCs) on day 1 (left), day 4 (middle) and day 9 (right). Scale bars: 275 µm (left and middle) and 650 µm (right).

Cell growth in the MC-based cultivations was mainly restricted by the growth surface. Figure 5a,b shows fluorescence microscopic pictures of DAPI-stained hASCs on MCs during cultivation in the spinner flasks. It is clear that on day 1 (after the cell attachment phase) nearly all of the MCs were covered by 2–5 cells. On day 4, some of the MCs were already partially covered with cells and the cells had started to form initial MC-cell-aggregates. By the end of the cultivation, almost all of the MCs were part of a MC-cell-aggregate and only a few MCs were floating around as single beads.

**Figure 6:**
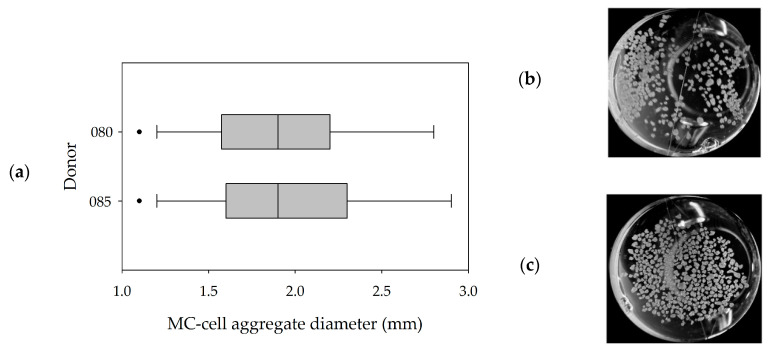
MC-cell-aggregate diameter distributions (**a**) and photographic images (**b**,**c**) of MC-cell aggregates at the end of the cultivations (day 9). Donor 080 (**b**) and 085 (**c**).

Figure 6a,b shows the MC-cell-aggregates at the end of the cultivations (=day 10) and the results of the size distribution analysis of the maximum MC-cell-aggregate diameters. It can be seen that a comparable MC-cell-aggregate size distribution was obtained at the end of both cultivations, with mean MC-cell-aggregate diameters of 1.95 mm (085) and 1.97 mm (080). Minimum and maximum MC-cell-aggregate diameters were measured at 0.8 mm and 5.7 mm for donor 080 and 0.7 mm and 5.0 mm for donor 085. This indicates local volume-weighted hydrodynamic stresses (*τ_nt_* = 4.96 × 10^−3^ Pa, *τ_nn_* = 1.15 × 10^−3^ Pa, Jossen et al. [1]) acting on the MC-cell-aggregates controlled their size to some extent. This observation also agreed well with literature findings [40,41]. Furthermore, the results indicated that increased MC-cell-aggregation mainly took place during the stationary growth phase. Therefore, the cell harvest point should also be defined based on MC-cell-aggregate size data.

### 3.4. Flow Cytometric Analysis of Standard Markers Expressed by hASCs Cultured in 2D or 3D

**Figure 7:**
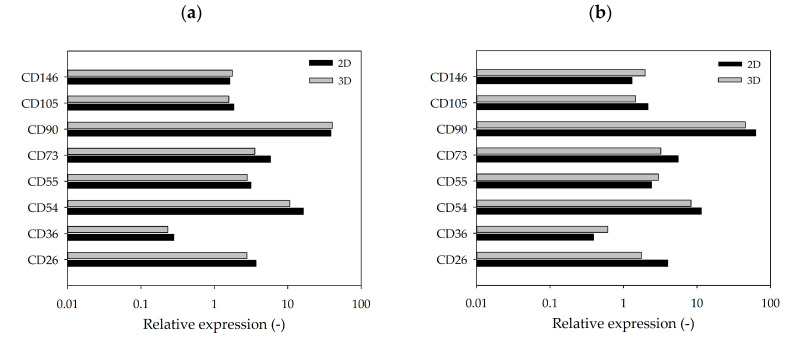
Flow cytometry expression profile of selected markers. hASCs from donor 080 (**a**) and donor 085 (**b**) cultivated in 2D (T_25_-flasks) or 3D (MC). hASCs were analyzed after harvesting on day 10. Mean fluorescence was calculated based on specific isotype controls (=relative marker expression).

Figure 7a,b shows the flow cytometry expression profiles of selected markers analyzed at the end of the cultivation studies (2D vs. 3D). There was no significant difference between the marker expression profile of cells cultured in 2D and those cultured in 3D on MCs. Moreover, no significant differences in the expression profiles were observable between the two donors, which agreed with our expectations. Positive hASC markers (CD26^+^, CD54^+^, CD55^+^, CD73^+^ and CD90^+^) were strongly expressed, while negative markers (CD36^−^ and CD146^−^) were only weakly expressed. CD105 was the only positive marker that was weakly expressed, which might be caused by the hyperconfluence of the cells after 10 days of cultivation [42,43,44].

### 3.5. Monitoring the Expression of Selected Stemness or Cell Differentiation Genes Measured by RT-qPCR

The cells used in this study were extracted from subcutaneous adipose tissue. It is therefore logical to assume that the “default differentiation pathway” of hASCs, in the event of unwanted and uncontrolled spontaneous maturation, is towards adipogenesis. In recent years, several important genes have been discovered that are crucial for the maintenance of stemness and for the differentiation of hASCs [34,45,46]. Further information about the different genes, including their relationships to each other and a short description, can be found in “Supplementary Materials” or in the literature [47,48,49,50,51,52,53,54,55,56,57,58,59,60,61,62,63,64,65,66,67,68,69,70,71,72,73,74,75,76,77,78,79,80,81,82,83]. The selected genes can be used as markers in RT-qPCR tests to assess and compare the differentiation status of hASCs expanded in static 2D (see Section 3.2) and/or dynamic 3D conditions (see Section 3.3). To facilitate analysis, the genes were subdivided into three groups:

A. Stemness maintenance genes: *PREF-1, SOX-9, ZFP521, WISP2, NOTCH1 and DLL1*
B. Differentiation regulators/markers: *PPARγ, ZFP423, RUNX2, DKK1, CD34, CD36, CD146* and *WISP1*
C. Lineage hierarchy markers: *CD26, CD55, CD142* and *CD248*

**Figure 8:**
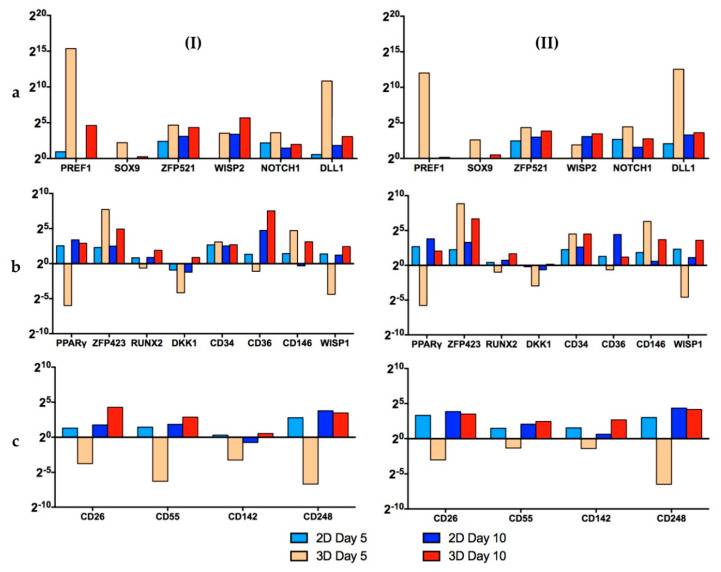
Results of RT-qPCR measurements of donors 080 (I) and 085 (II). The investigated genes were subdivided into 3 groups: (**a**) stemness maintenance genes, (**b**) differentiation regulators/markers and (**c**) lineage hierarchy markers. Data are represented as 2^(-ΔΔCt)^ and related to the beginning of the culture (day 1). A single value for each experimental condition was calculated with this method. This figure is also depicted as “heat maps” and is shown in the “Supplementary Materials” (Figure S3).

Figure 8 shows the results of the RT-qPCR measurements. In the category, “Stemness Maintenance” (Figure 8a), the cells of both donors on day 5 of the dynamic 3D cultivations had a better profile (high expression of *Pref-1* and *ZFP521*) than the hASCs grown in static 2D conditions. However, after 10 days, the cells were hyperconfluent in both cases. Consequently, the expression of almost all genes decreased [42,43,44]. Nonetheless, the cells on the MCs performed well and their gene expression pattern was similar to that of those obtained from hASCs grown in static 2D conditions.

In the second category, “Differentiation Regulators/Markers” (Figure 8b), it is clear that for the 3D dynamic conditions, the expression of *PPARγ*, *RUNX2*, *DKK1*, *CD34*, *CD36* and *WISP1* on day 5 was lower or very similar to the standard 2D set-up. Thus, it can be concluded that the cells retained their “stemness” under the dynamic 3D conditions. As already mentioned, on day 10, the cells were hyperconfluent in both cases. In this situation it is normal that the stemness genes are downregulated, while the differentiation genes are induced [42,44]. However, it is worth noting that the expression of *PPARγ*, the master regulator of adipogenesis, was lower for both donors (080, 085) under dynamic 3D conditions.

In the last category, “Lineage Hierarchy Markers” (Figure 8c), on day 5, the hASCs grown in standard 2D conditions displayed a better gene expression profile than the cells in 3D. However, on day 10, both cell culture systems showed similar satisfactory profiles. It should be noted that the expression of the four genes tested increased during the last days in the 3D spinner system, whereas in the 2D set-up it remained similar between days 5 and 10. 

The results of the RT-qPCR measurements clearly showed that the stemness of the hASCs was very well preserved when the cells were grown on xeno-free polystyrene-based MCs with the serum-free UrSuppe stem cell culture medium. The differences in the gene expression profiles were more pronounced on day 5. However, on day 10, due to the very high cell density, most of the stemness genes decreased and most of the differentiation genes increased in both systems, resulting in similar profiles [42,43,44]. These results clearly demonstrated that for both donors, the optimum point of harvest was on day 5–6 (see Section 3.3). This quality-related observation also agreed very well with the growth-related results and will have an influence on future growth investigations. Hence, higher MC amounts are required to provide the desired cell density, even at lower levels of cell confluency.

## 4. Conclusions

In this proof-of-concept study, growth- and quality-related investigations were performed under xeno- and serum-free conditions in planar 2D and dynamic 3D cultivation systems with hASCs isolated from two different patients/donors (080 and 085). In order to collect donor-dependent data, two donors of different ages (26 vs. 46 years) and with different health statuses (healthy and post-chemotherapy) were selected for this proof-of-concept study. The hASCs were isolated from the *SVF* under fully serum-free conditions in order to fulfill the regulatory requirements for future hASC manufacturing processes for autologous therapies. The results demonstrated that by using the serum-free UrSuppe stem cell culture medium, hASCs from both donors could be successfully isolated and cultured. The observed tissue frequency of living hASCs was comparable for both donors, although differences in age and health status existed. This is very important for future autologous therapies, as most patients/donors are older or unhealthy. However, further investigations in terms of the biological variation (e.g., gender, broad age range and health conditions) between donors and its effects on successful in-vitro cultivation at different production scales are necessary.

Growth characterization under static 2D conditions revealed differences in the growth performance and the maximum achievable cell densities for the two donors. Due to the higher mean cell areas for donor 085, maximum cell densities were lower, which reduced the overall total cell number per cultivation. The same observations were also performed during the MC-based cultivations. Information about cell morphology (i.e., cell size and area) and the maximum achievable cell density per donor under static 2D conditions is crucial for process scale-up in order to achieve the cell densities required for autologous therapies within the shortest time and number of passages (e.g., issue of cellular senescence). Due to the restricted maximum cell densities for individual donors, high amounts of MCs are necessary in order to increase the total cell number per process step. However, this has an effect on the choice of the stirred bioreactor type and the process conditions. As a result of the shear sensitivity of hASCs, the number of MCs can only be increased up to a certain point, where the occurring hydrodynamic stresses do not negatively affect cell growth or quality. The MC-based expansions clearly showed that the hydrodynamic stresses at *N_s1u_* did not significantly affect cell growth or cell quality, even though the stem cell culture medium did not contain FBS. The flow cytometric and RT-qPCR measurements highlighted the maintenance of the stemness during the static 2D and dynamic 3D cultivations of cells from both donors. Surface marker and gene expression profiles under dynamically mixed conditions were comparable for both donors and partially even better than for static 2D conditions. The results also clearly indicate that careful determination of the correct harvest point is important in order to retain stemness. A hyperconfluent culture would increase the total cell density per cultivation but lead to a downregulation of stemness maintenance genes and an upregulation of differentiation marker genes. Thus, optimal cell harvest densities for both donors were determined to be between 0.41 and 0.56 × 10^5^ hASCs/cm^2^, which were on average 14–22% lower than the maximum cell densities. Consequently, the required MC surface area per cultivation should in the future be defined based on the optimal cell harvest density.

The unstructured, segregated growth model very clearly showed time courses for cell growth, glucose consumption, lactate production and ammonium production that were similar to experimental data from the planar 2D and dynamic 3D cultivations. Maximum deviations for cell density and substrate/metabolite concentrations were in the range of 7–16% and 15–21%, respectively. This means that the descriptiveness power of the model was satisfactory, especially when considering the accuracy of the experimentally measured values. The intensified MC-cell-aggregate formation during the MC-based expansion was not considered in the growth model. Nevertheless, good agreement has been achieved. Therefore, the model can serve as basis for further investigations with hASCs. For this purpose, comprehensive growth studies with hASCs from a larger number of patient/donors (*n* = 12–20) are planned in stirred, instrumented single-use bioreactors (i.e., BioBLU 0.3c) based on a Design of Experiment approach.

## Figures and Tables

**Table 1:** Overview of primer sequences used for RT-qPCR measurements.

Genes	Forward Primer (5′-3′)	Reverse Primer (3′-5′)
*ACTB*	CTG GAA CGG TGA AGG TGA CA	AAG GGA CTT CCT GTA ACA ATG CA
*PREF1*	TGA CCA GTG CGT GAC CTC T	GGC AGT CCT TTC CCG AGT A
*SOX9*	AGC GAA CGC ACA TCA AGA C	CTG TAG GCG ATC TGT TGG GG
*WISP1*	CGA GGT ACG CAA TAG GAG TGT	GAA GGA CTG GCC GTT GTT GTA G
*WISP2*	GCG ACC AAC TCC ACG TCT G	TCC CCT TCC CGA TAC AGG C
*NOTCH1*	TGG ACC AGA TTG GGG AGT TC-3′	GCA CAC TCG TCT GTG TTG AC
*DLL1*	ACT CCG CGT TCA GCA ACC CCA T	TGG GTT TTC TGT TGC GAG GTC ATC AGG
*CD26*	AGT GGC ACG GCA ACA CAT T	AGA GCT TCT ATC CCG ATG ACT T
*CD55*	AGA GTT CTG CAA TCG TAG CTG C	CAC AAC AGT ACC GAC TGG AAA AT
*CD248*	AGT GTT ATT GTA GCG AGG GAC A	CCT CTG GGA AGC TCG GTC TA
*CD142*	GGC GCT TCA GGC ACT ACA A	TTG ATT GAC GGG TTT GGG TTC
*ZFP521*	GGC TGT TCA AAC ACA AGC G	GCA CAT TTA TAT GGC TTG TTG
*ZFP423*	GAT CAC TGT CAG CAG GAC TT	TGC CTC TTC AAG TAG CTC A
*PPARG*	TGA CAG CGA CTT GGC AAT ATT TAT T	TTG TAG CAG GTT GTC TTG AAT GTC T
*DKK1*	ATA GCA CCT TGG ATG GGT ATT CC	CTG ATG ACC GGA GAC AAA CAG
*RUNX2*	TCA ACG ATC TGA GAT TTG TGG G	GGG GAG GAT TTG TGA AGA CGG
*CD34*	TGG CTG TCT TGG GCA TCA CTG G	CTG AAT GGC CGT TTC TGG AGG TGG
*CD36*	TGT GCA AAA TCC ACA GGA AGT G	CCT CAG CGT CCT GGG TTA CA
*CD146*	AGC TCC GCG TCT ACA AAG C	CTA CAC AGG TAG CGA CCT CC

**Table 2:** Results obtained from the two different patients.

Donor	Heath Status	Region	Age	Live Cells	Live hASCs	hASCs
(-)	(-)	(-)	(-)	(10^6^ cells)	(10^5^ cells)	(%)
080	Healthy	Abdomen	46	9.5	5.4	5.7
085	Post-chemotherapy	Abdomen	26	4.8	3.7	7.7

**Table 3:** Overview of the main growth-dependent parameters in the 2D cultivations.

Donor	Xmax ^(^*^)^	PDL ^(^*^)^	EF ^(^**^)^	µ	t_d_	Y_Lac/Glc_	qGlc	qLac	qAmn
(-)	(10^5^ cells/cm^2^)	(-)	(-)	(d^−1^)	(h)	(mmol/mmol)	(pmol/cell/d)		
080	0.65 ± 0.02	3.22 ± 0.04	9.9	0.52	32.0	1.05	1.35	1.41	0.28
085	0.52 ± 0.02	2.79 ± 0.05	7.4	0.39	42.7	1.14	1.98	2.26	0.32

^(^*^)^ Value was calculated based on the values of the stationary growth phase (mean ± σ*_cells_*). (**) Value was calculated based on *X_min_* and *X_max_*.

**Table 4:** Overview of the main growth-dependent parameters in the Corning spinner flasks.

Donor	Xmax ^(^*^)^	PDL ^(^*^)^	EF ^(^**^)^	µ	t_d_	Y_Lac/Glc_	qGlc	qLac	qAmn
(-)	(10^5^ cells/cm^2^)	(-)	(-)	(d^−1^)	(h)	(mmol/mmol)	(pmol/cell/d)		
080	0.61 ± 0.01	1.58 ± 0.01	3.2	0.44	37.8	1.68	1.34	2.24	0.27
085	0.49 ± 0.01	1.72 ± 0.04	3.3	0.42	39.6	1.39	1.96	2.72	0.26

^(^*^)^ Value was calculated based on the values of the stationary growth phase (mean ± σ*_cells_*). ^(^**^)^ Value was calculated based on *X_min_* and *X_max_*.

## Supplementary Materials

The following are available online at https://www.mdpi.com/2306-5354/7/3/77/s1. Growth model principle and influencing factors in MC-based hASC expansions; Figure S1. Factors that positively or negatively regulate adipogenesis; Figure S2. Time-dependent profiles of cell density in the supernatant of the T25-flasks for donor 080 (a) and 085 (b); Figure S3. Results of RT-qPCR measurements (“heat maps”) of donors 080 (I) and 085 (II); Table S1. Parameters used for the kinetic growth model (2D and 3D); Table S2. Detailed information of the antibodies used for the flow cytometric measurements; Table S3. Reverse transcription detailed procedure; Table S4. Overview of measured stemness maintenance genes; Table S5. Overview of measured differentiation regulators/markers; Table S6. Overview of measured lineage hierarchy markers.

## Latin Symbols

*Amn*	mmol/L	Ammonium concentration
*EF*	-	Expansion factor
*Glc*	mmol/L	Glucose concentration
*k_at_*	d^−1^	Cell attachment constant
*k_det_*	d^−1^	Cell detachment constant
*K_Amn_*	mmol/L	Inhibition constant of ammonium
*K_Glc_*	mmol/L	Monod constant of glucose
*K_Lac_*	mmol/L	Inhibition constant of lactate
*Lac*	mmol/L	Lactate concentration
*N_s1u_*	rpm	Impeller speed at which the MCs are still in contact with the reactor bottom but none of the at rest (lower limit of *N_s1_*)
*PDL*	-	Population doubling level
*p_Amn_*	mmol/cell/d	Specific ammonium production rate (growth-independent)
*p_Lac_*	mmol/cell/d	Specific lactate production rate (growth-independent)
*qAmn*	mmol/cell/d	Specific ammonium production rate (growth-dependent)
*qGlc*	mmol/cell/d	Specific glucose consumption rate
*qLac*	mmol/cell/d	Specific lactate production rate (growth-dependent)
*t_d_*	d	Doubling time of cell population
*t_l_*	d	Lag or cell adaption time
*X_A_*	cells/cm^2^	Cell concentration on planar growth surface
*X_max_*	cells/cm^2^	Maximum cell concentration on planar growth surface
*X_Sus_*	cells/mL	Cell concentration in suspension
*X_V_*	cells/cm^2^	Cell concentration of viable cells (*X_Sus_ + X_A_*)
*Y_Lac/Glc_*	mmol/mmol	Lactate yield per glucose equivalent

## Greek Symbols

*α*	-	Cell adaption phase coefficient
*β_MC_*	g/L	Microcarrier concentration
*δ_Glc_*	-	Step response in glucose balance to avoid negative glucose values (*δ_Glc_* = 0 or 1)
*µ*	1/d	Specific cell growth rate
*µ_max_*	1/d	Maximum specific cell growth rate
*σ_cells_*	cells/cm^2^	Standard deviation of cell density
