# Supplementary material for: An Approach towards a GMP Compliant In-Vitro Expansion of Human Adipose Stem Cells for Autologous Therapies
Source: Bioengineering (Basel). 2020 Jul 20;7(3):77. doi: 10.3390/bioengineering7030077 (PMC7552624; doi:10.3390/bioengineering7030077)
Supplement: Supplementary file 1 [file bioengineering-07-00077-s001.pdf]

bioengineering

Additional information on the kinetic growth model (MC-based hASC growth):

The MC-based hASC growth in the Corning spinner flask was adapted from Jossen et al. [1] and divided into four steps (**I** cell sedimentation and initial cell attachment, **II** cell spreading and migration, **III** mitotic cell division and **IV** cell growth arrest due to contact inhibition), which partially ran in parallel (see Figure S1). During the cultivation period, the formation of MC-cell-aggregates was promoted due to the increasing number of cells per bead and periodic particle interactions. The rate of the MC-cell-aggregation was influenced by the frequency and strength of hydrodynamic stresses. However, the rate of MC-cell-aggregate formation was not considered in the current version of the MC-based growth model. All mathematical formulations used for simulating the MC-based hASC growth were comparable to those used for the 2D growth simulations.

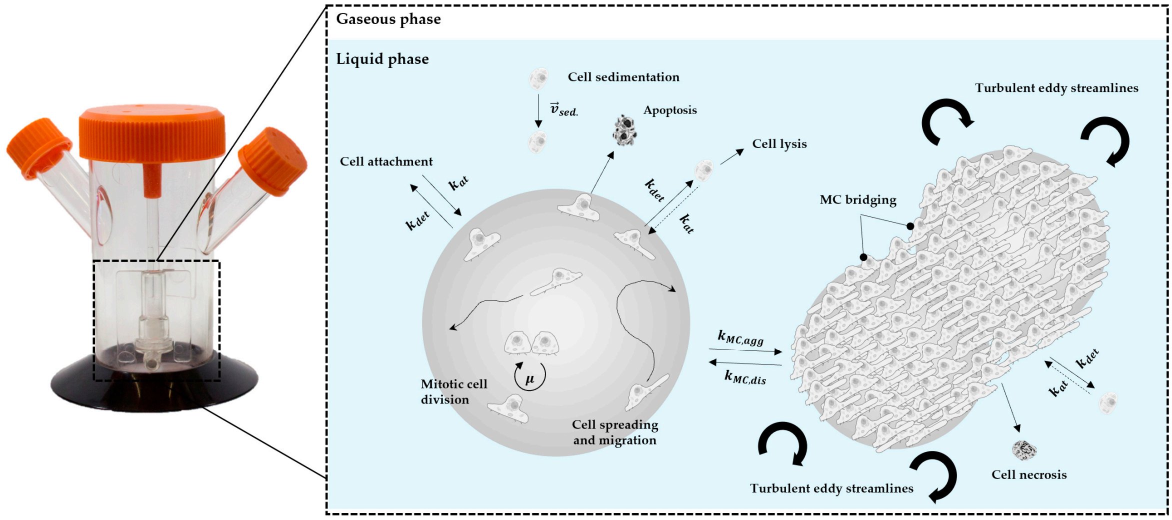

Figure S1. Growth model principle and influencing factors in MC-based hASC expansions (adapted from Jossen et al. [1]).

Table S1 summarizes the parameters used for the hASC growth simulations of the planar (2D) and MC-based (3D) cultivations. Metabolic flux and cell attachment/detachment parameters were used from this study. Affinity constants were adapted from the literature.

Table S1. Parameters used for the kinetic growth model (2D and 3D).

**Parameter**		**Value**	**Reference**
q_{Amn}	[pmol/cell/d]	0.26-0.32	This study
$-q_{Glc}$	[pmol/cell/d]	1.34-1.98	This study
q_{Lac}	[pmol/cell/d]	1.41-2.72	This study
k_{at}	[d^{-1}]	up to 50	This study
k_{det}	[d^{-1}]	up to 0.01	This study
K_{Amn}	[mmol/L]	8.0	Möhler et al. [2], Schop et al. [3]
K_{Glc}	[mmol/L]	0.4	Möhler et al. [2], Schop et al. [3]
K_{Lac}	[mmol/L]	105	Möhler et al. [2], Schop et al. [3]

Additional information for flow cytometric analysis:

Table S2 provides an overview of the antibodies and the concentrations used for flow cytometric measurements during *SVF* extraction and hASC processing.

Table S2. Detailed information of the antibodies used for the flow cytometric measurements.

Antibody	*Concentration for SVF [ng/µL]*	*Concentration for ASC [ng/µL]*	*# Catalog no.*	*Company*
CD26-FITC	-	50.0	302704	BioLegend
CD34-BV650	125.0	-	343624	BioLegend
CD36-APC	55.0	50.0	130-095-475	Miltenyi Biotec
CD45-PC7	125.0	-	304016	BioLegend
CD54-PE	-	50.0	12-0549-42	Thermo Fisher
CD55-BV421	-	50.0	742677	Becton Dickinson
CD73-FITC	75.0	50.0	344016	BioLegend
CD90-APC	-	50.0	328114	BioLegend
CD105-PE	-	50.0	323206	BioLegend
CD146-PE	34.0	50.0	130-092-853	Miltenyi Biotec
Syto40	1 µL	-	S11351	Thermo Fisher
7-AAD	2.5 µL	-	559925	Becton Dickinson
Zombie Yellow™	-	1:1000	423103	BioLegend

Additional information on the RT-qPCR analysis:

Table S3 summarizes the reagents and the respective amounts of each reagent used for the reverse transcription of the extracted RNA.

Table S3. Reverse transcription detailed procedure.

**Reagent**	**Amount**
**Mix 1:**	
RNA	Up to 2 µg
Oligo dT	0.5 µg
Random Primers	0.5 µg
H_2O	Final volume 5.0 µL
**Mix 2:**	
Buffer 5x	2.0 µL
$MgCl_2$	1.0 µL [2.5 mM]
dNTPs	0.5 µL [0.5 mM]
Inhibitor RNasi	0.25 µL [20 Units]
RT Enzyme	0.5 µL
H_2O	Final volume 5.0 µL
**Procedure:**	
Add Mix 1: incubate 5′ at 70 °C, cool to 10 °C and incubate 5′ in ice	
Add Mix 2: incubate 5′ at 25 °C, 42 °C for 1h and 70 °C for 15′	

Figure S2 schematically shows the relationship between different factors that positively or negatively regulate adipogenesis. The expression of the main regulation factors was measured in this study by RT-qPCR.

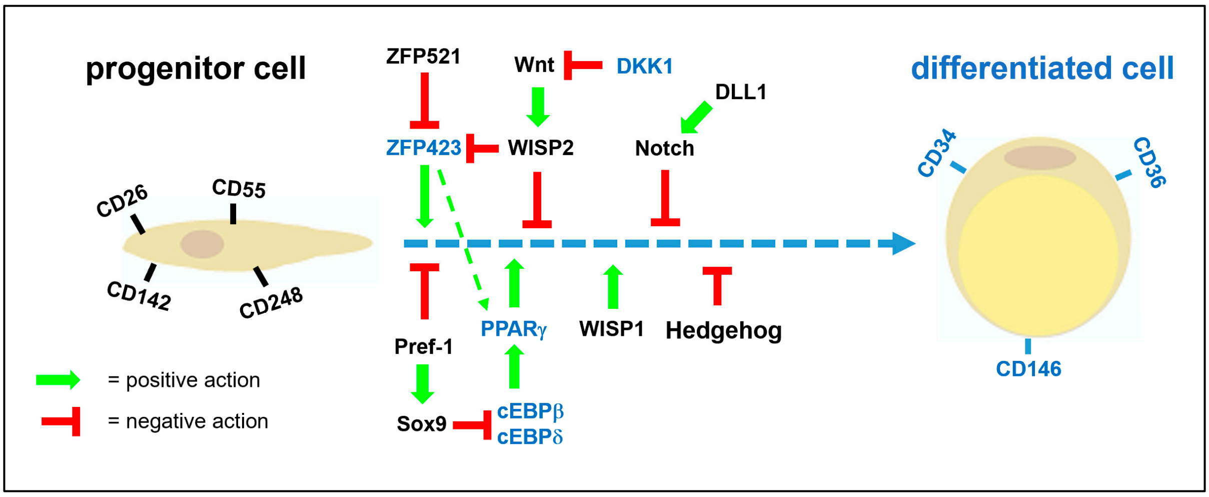

Figure S2. Factors that positively or negatively regulate adipogenesis. The blue dotted line represents the various stages of differentiation that lead to a mature adipocyte starting from a progenitor cell.

Table S4 provides an overview and short description of the stemness maintenance genes measured in this study.

Table S4. Overview of measured stemness maintenance genes.

Name	Description	Reference
PREF-1 (DIk-1)	Preadipocyte factor (Delta-like 1 homolog) is a transmembrane protein which inhibits adipogenesis and it belongs to the non-canonical Notch ligands family.	Hudak et al. [4]<br>Hei et al. [5]
SOX9	Sox9 is a member of the HMG-box class DNA-binding proteins and is a Pref-1 target.	Wang et al. [6]
ZFP525	Zinc Finger Protein 525 is a transcription factor which inhibits adipogenesis.	Chiarella et al. [7]<br>Kang et al. [8]
WISP2	Wnt-1 inducible signaling pathway protein 2 is an endogenous/secreted auto/paracrine non-conventional WNT ligand.	Grünberg et al. [9]<br>Hammarstedt et al. [10]
NOTCH1	It regulates the proliferation/differentiation of the adipocyte progenitor cells.	Shan et al. [11]<br>Ross et al. [12]
DLL1	Delta-like protein 1 is one of the five canonical Notch ligands. It inhibits adipogenesis.	Murata et al. [13]<br>Sparling et al. [14]

Table S5 provides an overview and short description of the differentiation regulators/markers measured in this study.

Table S5. Overview of measured differentiation regulators/markers.

Name	Description	Reference
PPARγ	Peroxisome Proliferator-Activated Receptor gamma is a ligand dependent transcription factor that is a member of the nuclear hormone receptor superfamily. It plays a crucial role in adipose tissue development/differentiation	Ahmadian et al. [15]<br>Barak et al. [16]<br>Rosen et al. [17]<br>Tontonoz et al. [18]
ZFP423	Zinc Finger Protein 423 is responsible for adipogenic commitment. It induces PPARγ expression & terminal adipogenic differentiation	Gupta et al. [19]<br>Gupta et al. [20]
RUNX2	Runx2 is a transcription factor that is essential for osteoblast differentiation and chondrocyte maturation	Komori et al. [21]
WISP1	The Wnt1-inducable signaling pathway protein-1 increases during adipocyte differentiation, stimulating adipogenesis	Ferrand et al. [22]<br>Murahovschi et al. [23]
DKK1	Dickkopf1 inhibits Wnt signaling and promotes differentiation	Christodoulides et al. [24]<br>Gustafson et al. [25]
CD34	CD34 is a transmembrane phosphorglycoprotein expressed on precursors cells and on mature adipocytes. Its function on the adipocyte membrane is still to be determined	Festy et al. [26]<br>Sidney et al. [27]<br>Scherberich et al. [28]
CD36	CD36 is a transmembrance glycoprotein classified as a class B scavenger receptor. It plays a functional role in adipocyte differentiation/adipogenesis	Christiaens et al. [29]<br>Gao et al. [30]
CD146	Three forms of this adhesion protein have been described, including two transmembrane isoforms and a soluble protein, detectable in the plasma. Its expression increases during adipogenic differentiation	Leroyer et al. [31]<br>Walmsley et al. [32]

Table S6 provides an overview and short description of the lineage hierarchy markers measured in this study.

Table S6. Overview of measured lineage hierarchy markers.

Name	Description	Reference
CD26	Dipeptdyl peptidase-4 (DPP4), also known as adenosine deaminase complexing protein 2 or CD26. It is associated with immune regulation, signal transduction & apoptosis	Mortier et al. [33]<br>Metzemaekers et al. [34]<br>Merrick et al. [35]<br>Rennert et al. [36]
CD55	Complement decay-accelerating factor, also known as CD55 or DAF, is a protein that, in humans, regulates the complement system on the cell surface	Merrick t al. [35]<br>Rennert et al. [36]
CD142	Tissue factor, also called platelet tissue factor, factor III, or CD142, is a protein present in subendothelial tissue. It is the primary inhibitor of the blood coagulation cascade	Schwalie et al. [37]<br>Chu et al. [38]
CD248	CD248, also known as endosialin and tumor endothelial marker 1 (TEM-1). This marker does not have a fully characterized role, but its expression has been associated with angiogenesis in the embryo and uterus as well as in tumor development and growth.	Brett et al. [39]<br>Merrick et al. [35]

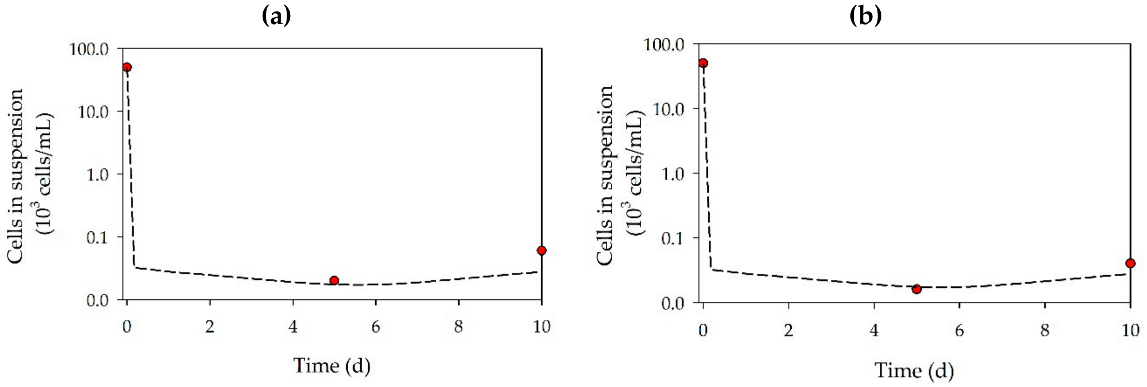

Figure S3. Time-dependent profiles of cell density in the supernatant of the T_{25}-flasks for donor 080 (a) and 085 (b). The symbols represent the experimentally measured values collected from offline measurements. The lines represent the simulated time courses.

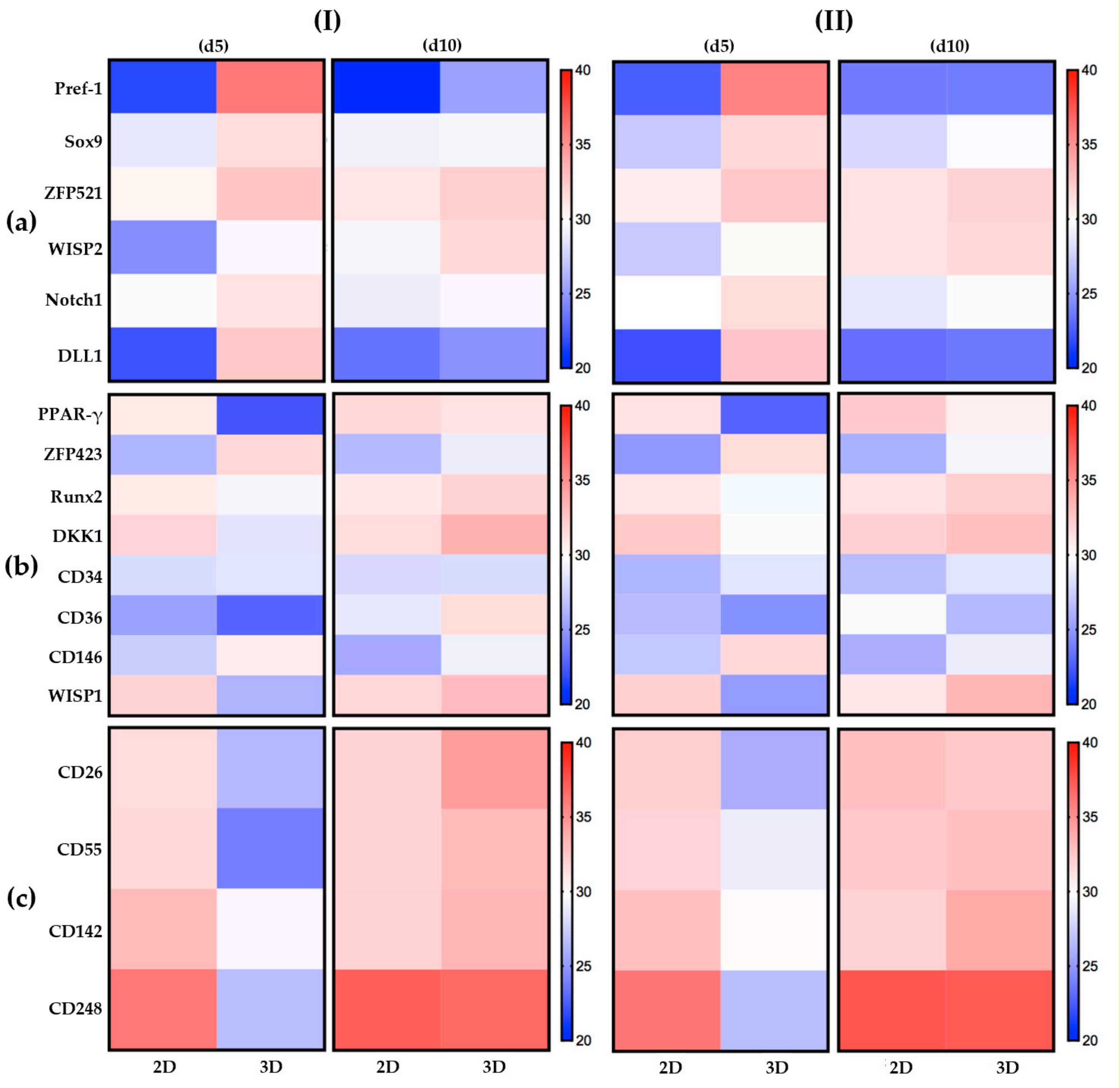

Figure S4. Results of RT-qPCR measurements ("heat maps") of donors 080 (I) and 085 (II). The investigated genes were subdivided into 3 groups: (a) Stemness Maintenance genes, (b) Differentiation Regulators/Markers, (c) Lineage Hierarchy Markers. Colormap = relative gene expression 20 to 40-fold.

References Supplementary Materials

[1] V. Jossen, R. Eibl, M. Kraume, D. Eibl, Growth Behavior of Human Adipose Tissue-Derived Stromal/Stem Cells at Small Scale: Numerical and Experimental Investigations, Bioengineering. 5 (2018) 106. https://doi.org/10.3390/bioengineering5040106.

[2] L. Möhler, A. Bock, U. Reichl, Segregated mathematical model for growth of anchorage-dependent MDCK cells in microcarrier culture, Biotechnol. Prog. 24 (2008) 110–119. https://doi.org/10.1021/bp0701923.

[3] D. Schop, F.W. Janssen, L.D. van Rijn, H. Fernandes, R.M. Bloem, J.D. de Bruijn, R. van Dijkhuizen-Radersma, Growth, metabolism, and growth inhibitors of mesenchymal stem cells, Tissue Eng. Part A. 15 (2009) 1877–1886. https://doi.org/10.1089/ten.tea.2008.0345.

[4] C.S. Hudak, O. Gulyaeva, Y. Wang, S.M. Park, L. Lee, C. Kang, H.S. Sul, Pref-1 marks very early mesenchymal precursors required for adipose tissue development and expansion, Cell Rep. 8 (2014) 678–687.

[5] H.S. Sul, Minireview: Pref-1: Role in Adipogenesis and Mesenchymal Cell Fate, Mol. Endocrinol. 23 (2009) 1717–1725. https://doi.org/10.1210/me.2009-0160.

[6] Y. Wang, H.S. Sul, Pref-1 Regulates Mesenchymal Cell Commitment and Differentiation through Sox9, Cell Metab. 9 (2009) 287–302. https://doi.org/10.1016/j.cmet.2009.01.013.

[7] E. Chiarella, A. Aloisio, B. Codispoti, G. Nappo, S. Scicchitano, V. Lucchino, Y. Montalcini, A. Camarotti, O. Galasso, M. Greco, G. Gasparini, M. Mesuraca, H.M. Bond, G. Morrone, ZNF521 Has an Inhibitory Effect on the Adipogenic Differentiation of Human Adipose-Derived Mesenchymal Stem Cells, Stem Cell Rev. Reports. 14 (2018) 901–914. https://doi.org/10.1007/s12015-018-9830-0.

[8] S. Kang, P. Akerblad, R. Kiviranta, R.K. Gupta, S. Kajimura, M.J. Griffin, J. Min, R. Baron, E.D. Rosen, Regulation of early adipose commitment by Zfp521, PLoS Biol. 10 (2012).

[9] J.R. Grünberg, J. Elvin, A. Paul, S. Hedjazifar, A. Hammarstedt, U. Smith, CCN5 / W ISP2 and Metabolic Diseases, (2018) 309–318.

[10] A. Hammarstedt, S. Hedjazifar, L. Jenndahl, S. Gogg, J. Grunberg, B. Gustafson, E. Klimcakova, V. Stich, D. Langin, M. Laakso, U. Smith, WISP2 regulates preadipocyte commitment and PPAR activation by BMP4, Proc. Natl. Acad. Sci. 110 (2013) 2563–2568. https://doi.org/10.1073/pnas.1211255110.

[11] D. Ross, R. Prakash, T. Kadesch, Dual Roles for the Notch Target Gene Hes-1 in the Differentiation of 3T3-L1 Preadipocytes, 24 (2004) 3505–3513. https://doi.org/10.1128/MCB.24.8.3505.

[12] T. Shan, J. Liu, W. Wu, Z. Xu, Y. Wang, Roles of Notch Signaling in Adipocyte Progenitor Cells and Mature Adipocytes, J. Cell. Physiol. 232 (2017) 1258–1261. https://doi.org/10.1002/jcp.25697.

[13] D.P. Sparling, J. Yu, K. Kim, C. Zhu, S. Brachs, A.L. Birkenfeld, U.B. Pajvani, Adipocyte-specific blockade of gamma-secretase, but not inhibition of Notch activity, reduces adipose insulin sensitivity, Mol. Metab. 5 (2016) 113–121. https://doi.org/10.1016/j.molmet.2015.11.006.

[14] A. Murata, M. Yoshino, M. Hikosaka, K. Okuyama, L. Zhou, S. Sakano, H. Yagita, S.-I. Hayashi, An Evolutionary-Conserved Function of Mammalian Notch Family Members as Cell Adhesion Molecules, PLoS One. 9 (2014) e108535. https://doi.org/10.1371/journal.pone.0108535.

[15] M. Ahmadian, J.M. Suh, N. Hah, C. Liddle, A.R. Atkins, M. Downes, R.M. Evans, PPARγ signaling and metabolism: the good, the bad and the future, Nat. Med. 19 (2013) 557–566. https://doi.org/10.1038/nm.3159.

[16] Y. Barak, M.C. Nelson, E.S. Ong, Y.Z. Jones, P. Ruiz-Lozano, K.R. Chien, A. Koder, R.M. Evans, PPARγ Is Required for Placental, Cardiac, and Adipose Tissue Development, Mol. Cell. 4 (1999) 585–595. https://doi.org/10.1016/S1097-2765(00)80209-9.

[17] E.D. Rosen, P. Sarraf, A.E. Troy, G. Bradwin, K. Moore, D.S. Milstone, B.M. Spiegelman, R.M. Mortensen, PPARγ Is Required for the Differentiation of Adipose Tissue In Vivo and In Vitro, Mol. Cell. 4 (1999) 611–617. https://doi.org/10.1016/S1097-2765(00)80211-7.

[18] P. Tontonoz, E. Hu, B.M. Spiegelman, Stimulation of adipogenesis in fibroblasts by PPARγ2, a lipid-activated transcription factor, Cell. 79 (1994) 1147–1156. https://doi.org/10.1016/0092-8674(94)90006-X.

[19] R.K. Gupta, Z. Arany, P. Seale, R.J. Mepani, L. Ye, H.M. Conroe, Y.A. Roby, H. Kulaga, R.R.

Reed, B.M. Spiegelman, Transcriptional control of preadipocyte determination by Zfp423, Nature. 464 (2010) 619–623.

[20] R.K. Gupta, R.J. Mepani, S. Kleiner, J.C. Lo, M.J. Khandekar, P. Cohen, A. Frontini, D.C. Bhowmick, L. Ye, S. Cinti, B.M. Spiegelman, Zfp423 Expression Identifies Committed Preadipocytes and Localizes to Adipose Endothelial and Perivascular Cells, Cell Metab. 15 (2012) 230–239. https://doi.org/10.1016/j.cmet.2012.01.010.

[21] T. Komori, Runx2, an inducer of osteoblast and chondrocyte differentiation, Histochem. Cell Biol. 149 (2018) 313–323. https://doi.org/10.1007/s00418-018-1640-6.

[22] N. Ferrand, V. Béreziat, M. Moldes, M. Zaoui, A.K. Larsen, M. Sabbah, WISP1/CCN4 inhibits adipocyte differentiation through repression of PPARγ activity, Sci. Rep. 7 (2017) 1749. https://doi.org/10.1038/s41598-017-01866-2.

[23] V. Murahovschi, O. Pivovarova, I. Ilkavets, R.M. Dmitrieva, S. Döcke, F. Keyhani-Nejad, Ö. Gögebakan, M. Osterhoff, M. Kemper, S. Hornemann, M. Markova, N. Klöting, M. Stockmann, M.O. Weickert, V. Lamounier-Zepter, P. Neuhaus, A. Konradi, S. Dooley, C. von Loeffelholz, M. Blüher, A.F.H. Pfeiffer, N. Rudovich, WISP1 Is a Novel Adipokine Linked to Inflammation in Obesity, Diabetes. 64 (2015) 856–866. https://doi.org/10.2337/db14-0444.

[24] C. Christodoulides, The Wnt antagonist Dickkopf-1 and its receptors are coordinately regulated during early human adipogenesis, J. Cell Sci. 119 (2006) 2613–2620. https://doi.org/10.1242/jcs.02975.

[25] B. Gustafson, U. Smith, The WNT Inhibitor Dickkopf 1 and Bone Morphogenetic Protein 4 Rescue Adipogenesis in Hypertrophic Obesity in Humans, Diabetes. 61 (2012) 1217–1224. https://doi.org/10.2337/db11-1419.

[26] F. Festy, L. Hoareau, S. Bes-Houtmann, A.-M. Péquin, M.-P. Gonthier, A. Munstun, J.J. Hoarau, M. Césari, R. Roche, Surface protein expression between human adipose tissue-derived stromal cells and mature adipocytes, Histochem. Cell Biol. 124 (2005) 113–121. https://doi.org/10.1007/s00418-005-0014-z.

[27] L.E. Sidney, M.J. Branch, S.E. Dunphy, H.S. Dua, A. Hopkinson, Concise Review: Evidence for CD34 as a Common Marker for Diverse Progenitors, Stem Cells. 32 (2014) 1380–1389. https://doi.org/10.1002/stem.1661.

[28] A. Scherberich, A familiar stranger: CD34 expression and putative functions in SVF cells of adipose tissue, World J. Stem Cells. 5 (2013) 1. https://doi.org/10.4252/wjsc.v5.i1.1.

[29] V. Christiaens, M. Van Hul, H.R. Lijnen, I. Scroyen, CD36 promotes adipocyte differentiation and adipogenesis, Biochim. Biophys. Acta - Gen. Subj. 1820 (2012) 949–956. https://doi.org/10.1016/j.bbagen.2012.04.001.

[30] H. Gao, F. Volat, L. Sandhow, J. Galitzky, T. Nguyen, D. Esteve, G. Åström, N. Mejhert, S. Ledoux, C. Thalamas, P. Arner, J.-C. Guillemot, H. Qian, M. Rydén, A. Bouloumié, CD36 Is a Marker of Human Adipocyte Progenitors with Pronounced Adipogenic and Triglyceride Accumulation Potential, Stem Cells. 35 (2017) 1799–1814. https://doi.org/10.1002/stem.2635.

[31] A.S. Leroyer, M.G. Blin, R. Bachelier, N. Bardin, M. Blot-Chabaud, F. Dignat-George, CD146 (Cluster of Differentiation 146): An Adhesion Molecule Involved in Vessel Homeostasis, Arterioscler. Thromb. Vasc. Biol. 39 (2019) 1026–1033. https://doi.org/10.1161/ATVBAHA.119.312653.

[32] G.G.G. Walmsley, D.A.D.A. Atashroo, Z.N.Z.N.Z.N. Maan, M.S.M.S. Hu, E.R.E.R.E.R. Zielins,

J.M.J.M. Tsai, D. Duscher, K. Paik, R. Tevlin, O. Marecic, D.C.D.C. Wan, G.C.G.C. Gurtner, M.T.M.T. Longaker, High-Throughput Screening of Surface Marker Expression on Undifferentiated and Differentiated Human Adipose-Derived Stromal Cells, Tissue Eng. Part A. 21 (2015) 2281–2291. https://doi.org/10.1089/ten.tea.2015.0039.

[33] A. Mortier, M. Gouwy, J. Van Damme, P. Proost, S. Struyf, CD26/dipeptidylpeptidase IV-chemokine interactions: double-edged regulation of inflammation and tumor biology, J. Leukoc. Biol. 99 (2016) 955–969. https://doi.org/10.1189/jlb.3MR0915-401R.

[34] M. Metzemaekers, J. Van Damme, A. Mortier, P. Proost, Regulation of Chemokine Activity – A Focus on the Role of Dipeptidyl Peptidase IV/CD26, Front. Immunol. 7 (2016). https://doi.org/10.3389/fimmu.2016.00483.

[35] D. Merrick, A. Sakers, Z. Irgebay, C. Okada, C. Calvert, M.P. Morley, I. Percec, P. Seale, Identification of a mesenchymal progenitor cell hierarchy in adipose tissue, Science (80-.). 364 (2019) eaav2501. https://doi.org/10.1126/science.aav2501.

[36] R.C. Rennert, M. Januszyk, M. Sorkin, M. Rodrigues, Z.N. Maan, D. Duscher, A.J. Whittam, R. Kosaraju, M.T. Chung, K. Paik, A.Y. Li, M. Findlay, J.P. Glotzbach, A.J. Butte, G.C. Gurtner, Microfluidic single-cell transcriptional analysis rationally identifies novel surface marker profiles to enhance cell-based therapies, Nat. Commun. 7 (2016) 11945. https://doi.org/10.1038/ncomms11945.

[37] P.C. Schwalie, H. Dong, M. Zachara, J. Russeil, D. Alpern, N. Akchiche, C. Caprara, W. Sun, K.-U. Schlaudraff, G. Soldati, C. Wolfrum, B. Deplancke, A stromal cell population that inhibits adipogenesis in mammalian fat depots, Nature. 559 (2018) 103–108. https://doi.org/10.1038/s41586-018-0226-8.

[38] A.J. Chu, Tissue Factor, Blood Coagulation, and Beyond: An Overview, Int. J. Inflam. 2011 (2011) 1–30. https://doi.org/10.4061/2011/367284.

[39] E. Brett, E.R. Zielins, M. Chin, M. Januszyk, C.P. Blackshear, M. Findlay, A. Momeni, G.C. Gurtner, M.T. Longaker, D.C. Wan, Isolation of CD248-expressing stromal vascular fraction for targeted improvement of wound healing, Wound Repair Regen. 25 (2017) 414–422. https://doi.org/10.1111/wrr.12542.

©2020 by the authors. Licensee MDPI, Basel, Switzerland. This article is an open access article distributed under the terms and conditions of the Creative Commons Attribution (CC BY) license (http://creativecommons.org/licenses/by/4.0/).
